# Polydisperse molecular architecture of connexin 26/30 heteromeric hemichannels revealed by atomic force microscopy imaging

**DOI:** 10.1074/jbc.RA119.012128

**Published:** 2021-01-13

**Authors:** Pamela A. Naulin, Benjamin Lozano, Christian Fuentes, Yu Liu, Carla Schmidt, Jorge E. Contreras, Nelson P. Barrera

**Affiliations:** 1 Department of Physiology, Faculty of Biological Sciences, Pontificia Universidad Católica de Chile, Santiago, Chile; 2 Department of Pharmacology, Physiology and Neuroscience, New Jersey Medical School, Rutgers University, Newark, New Jersey, USA; 3 Department of Chemistry, University of Oxford, Oxford, United Kingdom

**Keywords:** atomic force microscopy, Fab fragment, statistics, stoichiometry, subunit arrangement, membrane protein, connexin, heteromer, AFM

## Abstract

Connexin (Cx) protein forms hemichannels and gap junctional channels, which play diverse and profound roles in human physiology and diseases. Gap junctions are arrays of intercellular channels formed by the docking of two hemichannels from adjacent cells. Each hexameric hemichannel contains the same or different Cx isoform. Although homomeric Cxs forms have been largely described functionally and structurally, the stoichiometry and arrangement of heteromeric Cx channels remain unknown. The latter, however, are widely expressed in human tissues and variation might have important implications on channel function. Investigating properties of heteromeric Cx channels is challenging considering the high number of potential subunit arrangements and stoichiometries, even when only combining two Cx isoforms. To tackle this problem, we engineered an HA tag onto Cx26 or Cx30 subunits and imaged hemichannels that were liganded by Fab-epitope antibody fragments via atomic force microscopy. For Cx26-HA/Cx30 or Cx30-HA/Cx26 heteromeric channels, the Fab-HA binding distribution was binomial with a maximum of three Fab-HA bound. Furthermore, imaged Cx26/Cx30-HA triple liganded by Fab-HA showed multiple arrangements that can be derived from the law of total probabilities. Atomic force microscopy imaging of ringlike structures of Cx26/Cx30-HA hemichannels confirmed these findings and also detected a polydisperse distribution of stoichiometries. Our results indicate a dominant subunit stoichiometry of 3Cx26:3Cx30 with the most abundant subunit arrangement of Cx26-Cx26-Cx30-Cx26-Cx30-Cx30. To our knowledge, this is the first time that the molecular architecture of heteromeric Cx channels has been revealed, thus providing the basis to explore the functional effect of these channels in biology.

Connexin (Cx) hemichannels are hexameric membrane proteins that can assemble as homomeric or heteromeric hemichannels. Each hemichannel can interact with a counterpart across an extracellular gap to form an intercellular gap junction (GJ) channel. These channels play essential roles in the cell-cell communication, providing both electric and metabolic coupling through the passive transport of ions and solutes (second messengers, amino acids, nucleotides, glucose) (1). The human proteome has 21 isoforms of Cx, and in general, almost all cell types, except for erythrocytes and sperm, express two or more Cxs.

There is substantial functional and structural diversity of hemichannels because of the number of Cx isoforms and the capacity to form heteromeric hemichannels. In theory, for 2 Cx isoforms there are 5 possible heteromeric stoichiometries and 11 possible arrangements within a hemichannel. With the 2 homomeric possibilities, a total of 13 different molecular architectures of hemichannel forms could exist (1), which challenges the application of common structural biology approaches. This diversity of Cx hemichannel assemblies provides cells with the ability to dynamically regulate their communication properties.

Heteromeric hemichannels have been reported in many types of tissues, including liver (2), lens (3), mammary glands (4), and inner ear (5). In lens, it has been well-documented that proper oligomerization of Cx46 and Cx50 is crucial for clarity and growth lens (6). In the cochlea, Cx26 and Cx30 are co-expressed (7), forming heteromeric hemichannels able to propagate a calcium signal twice as fast as its homomeric counterparts (5). On the other hand, permeability studies using HeLa cells co-transfected with Cx26 and Cx30 showed that the heteromeric channels only transport cations, unlike Cx26 homomeric channels, which can transport both cations and anions (8), providing further evidence that heteromeric channels exhibit different transport selectivity and biophysical properties. The high molecular selectivity that heteromeric channels can present was shown by Ayad *et al.* (9), who compared the permeability of homomeric (Cx26 or Cx32) and heteromeric (Cx26/Cx32) channels to different inositol phosphates. They demonstrated that heteromeric channels are highly selective, able to discriminate among different isomers of inositol phosphate, suggesting that this selective permeability is because of different heteromeric conformations (9).

Recently, the structure of native lens Cx46/Cx50 GJ channels has been resolved by cryo-EM; however, it was not possible to resolve the subunit arrangement of Cx46/50 heteromeric hemichannels or heterotypic GJ channels (10). A method based on atomic force microscopy (AFM) imaging has permitted resolution of the stoichiometry and subunit arrangement of several membrane proteins such as the GABA_A_ receptor (11), the P2X receptor (12, 13, 14, 15), the 5-HT3 receptor (16), the TRP channel (17, 18, 19, 20, 21, 22), the ASIC1a channel (23), the ENaC channel (24), the Kv7 channel (25), and the ionotropic glutamate receptor (26). Briefly, this method involves engineering specific epitope tags onto each subunit and expressing the proteins in a suitable cell line. Crude membrane fractions from transfected cells are solubilized in detergent, and tagged membrane proteins are purified, then imaged by AFM, and their mean molecular volume is compared with the molecular volume expected for the protein, based on its molecular weight. The proteins are incubated with antibodies to the tags, and the resulting multimer antibody complexes are imaged by AFM. Multimers with two or more bound antibodies are identified, and the angles between the antibodies are measured. The frequency distribution of these angles then reveals the architecture of the multimer (reviewed in Ref. 27).

In the present study, we used AFM imaging of heteromeric Cx26/Cx30 hemichannels decorated by Fab antibody fragments against subunit-specific epitope tags (Fab-epitope) to determine the subunit stoichiometry and arrangement. We show that co-expression of Cx26 and Cx30 yields purified heteromeric hemichannels with a dominant stoichiometry of 3:3 mainly arranged by 2×Cx26-Cx30-Cx26-2×Cx30. This was further corroborated by imaging ringlike structures of heteromeric Cx26/Cx30 hemichannels. To our knowledge, this is the first report showing the molecular architecture of Cx heteromeric hemichannels, which could have profound implications on their biophysical properties *versus* homomeric species.

## Results

### Stoichiometry of the Cx26/Cx30 hemichannels

**Figure 1:**
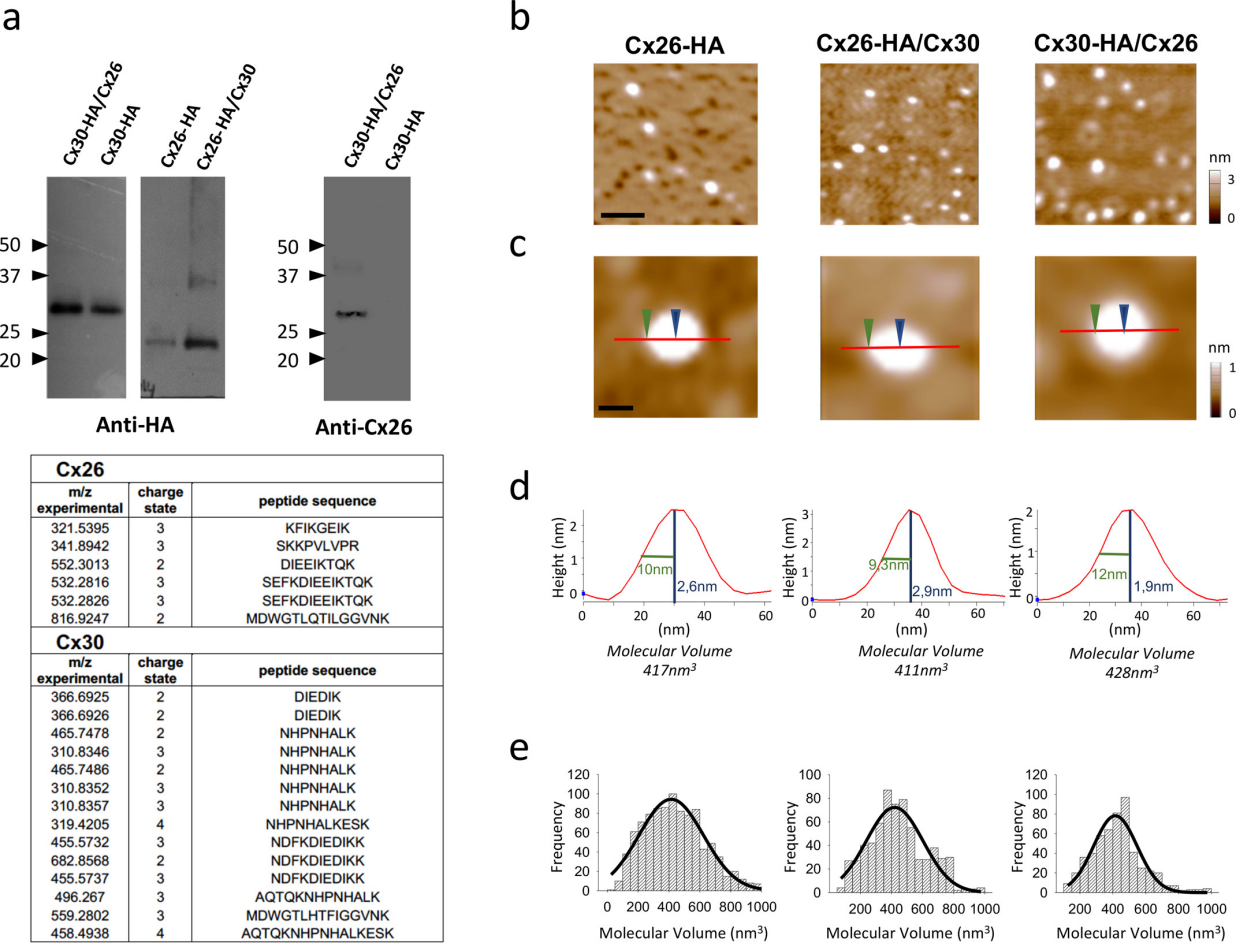
**Molecular volumes obtained for homomeric Cx26-HA, heteromeric Cx26-HA/Cx30, and Cx30-HA/Cx26 hemichannels from AFM imaging.***a*, identification of purified hemichannels. *Upper*, detection of Cx30-HA/Cx26, Cx26-HA/Cx30, and Cx26-HA protein in eluates from HA-agarose columns. Samples were analyzed by SDS-PAGE and immunoblotting, using mono and polyclonal anti-HA and anti-Cx26 primary Ab, respectively, followed by a horseradish peroxidase–conjugated goat anti-mouse and rabbit secondary Ab, respectively. *Lower*, peptide sequences identified by MS analysis after tryptic digestion from heteromeric Cx30-HA/Cx26 hemichannels, corresponding to sequences found for both Cx30 and Cx26 isoforms. *b*, low-magnification image (*scale bar*, 50 nm). *c*, high-magnification images of single proteins. Sections through particles are shown as *red lines* including two points, height and radius at half height (*blue* and *green arrows*, respectively) (*scale bar*, 20 nm). *d*, particle height analysis of the indicated section. *e*, frequency distribution of molecular volumes. *Black curves* indicate fitted Gaussian functions.

To confirm that the HA-affinity purification of Cx26/Cx30 hemichannels was successful, immunoblots were carried out with anti-HA and anti-Cx26 antibodies. For the anti-HA antibody, a band is expected at 34 kDa when the tag is on Cx30 and at 30 kDa when it is on Cx26 according to molecular weights deduced from the protein sequence. However, it has been observed that some Cx(s) migrate more rapidly on SDS-PAGE (Fig. 1*a*, *upper panel*) (28). It is important to note that the purified protein–antiCx26 antibody interaction is specific for Cx26, because the band was observed in heteromeric Cx30-HA/Cx26 but not in homomeric Cx30 hemichannels. Additionally, heteromeric Cx30-HA/Cx26 hemichannels were analyzed by liquid chromatography–coupled tandem MS after tryptic digestion, and peptides coinciding with the amino acid sequence of both Cx(s) (14 peptides for Cx30 and 6 peptides for Cx26, details in Table S1) confirmed the presence of the heteromers in the purified sample (Fig. 1*a*, *lower panel*).

After purification, homomeric and heteromeric hemichannels were adsorbed onto the mica surface and visualized by AFM to determine the molecular volume of the protein. As shown in Fig. 1*b*, the Cx26-HA, Cx26-HA/Cx30, and Cx30-HA/Cx26 hemichannels were observed as a population of particles homogenously spread. From those images, radii and heights of several hemichannel particles from each sample were measured (Fig. 1, *c* and *d*). Particle radii were measured at half the maximal height to compensate for the tendency of AFM to overestimate this parameter when the radii of particle and scanning tip are similar. By using this method, a very good correlation was obtained previously between theoretical and experimental molecular volumes for proteins of widely varying masses (29). Then, using the height and radii, the molecular volume for each particle was calculated by applying Equation 1 (see “Experimental procedures”). The histogram of molecular volumes obtained for each sample was fitted to a Gaussian function by using nonlinear regression (Fig. 1*e*). For the heteromeric hemichannels, molecular volumes of the particle population analyzed were centered at peaks of 420 ± 15 nm^3^ (*n* = 660) for Cx26-HA/Cx30, 415 ± 11 nm^3^ (*n* = 539) for Cx30-HA/Cx26 and for homomeric hemichannels the particles population analyzed were centered at peaks 414 ± 9 nm^3^ (*n* = 1000) for Cx26-HA and 390 ± 6 nm^3^ (*n* = 1000) for Cx30-HA (Fig. S1). In theory, if the molecular weight of a Cx26 plus tag is ∼30 kDa (Cx26-HA) and the hemichannel is made up of six subunits, the molecular weight of the hemichannel is 177 kDa. The expected molecular volume calculated from Equation 2 (see “Experimental procedures”) is 335 nm^3^, but the peak of molecular volume experimentally determined for homomeric Cx26-HA was greater than expected. The increase in experimental molecular volume is likely caused by the presence of detergent micelles around the transmembrane region of the proteins (Table S2). Then, for heteromeric Cx26-HA/Cx30 hemichannels containing one or more copies of Cx30 subunit, the expected molecular volume should be greater than homomeric Cx26-HA (Table S2). However, no significant differences (*p* > 0.05, unpaired *t* test) were obtained between homomeric and heteromeric Cx hemichannels via AFM imaging, which demonstrates that it is not possible to differentiate assembled hemichannels at single molecule level solely based on the molecular volume.

Hence to determine the stoichiometry of heteromeric Cx26/Cx30 hemichannels, these were decorated with anti-tag antibodies (anti-HA). By this approach, a significant difference between volumes of the purified protein and antibody can be obtained when the antibody is four times smaller than the tagged protein. Considering that the molecular volumes measured for the Cx hemichannels were around 400 nm^3^ (Fig. 1) and for anti-HA antibody 153 nm^3^ ± 1 nm^3^ (Fig. S1), this criterion is not achieved. Therefore, Fab-HA antibody fragments (Fab-HA) were used instead of full antibody. The molecular volume obtained for the Fab-HA was 105 nm^3^ ± 1 nm^3^ (Fig. S1), correlating well with their theoretical volume (94 nm^3^).

**Figure 2:**
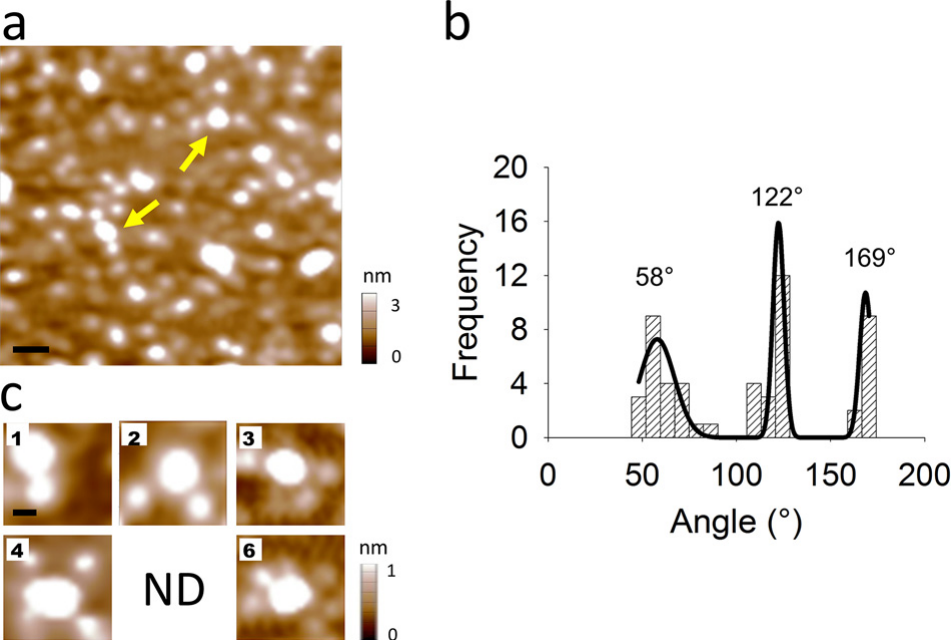
**Homomeric Cx26-HA hemichannels decorated with Fab-HA.***a*, AFM imaging of co-incubated Cx26-HA hemichannels with Fab-HA. *Yellow arrows* show double Fab-HA binding (*scale bar*, 50 nm). *b*, frequency distribution of angles from double Fab-HA binding. *c*, gallery of images showing the different number (indicated in the *left corner*) of Fab-HA bound to Cx26-HA hemichannels (scale bar, 20 nm). *ND*, not detected.

As control experiments, first Fab-HA decoration of the homomeric hemichannel Cx26-HA was carried out. As expected for a sequential and nonsterically dependent process of binding, the number of multiple Fab-HA binding decays in a binomial probability distribution (Equations 3 and 4, see “Experimental procedures”). This has been observed in a variety of membrane protein decorations (11, 12, 15, 16, 26) with anti-subunit (or tag) antibodies. In consequence, the efficiency of the theoretical binomial binding process for the homomeric Cx26-HA hemichannel could reach up to only 1.4% above three multiple bindings, which is close to 1.0% determined in our experiments (Table 1), suggesting that our methodology is able to detect these multiple binding events. Representative single, double, triple, quadruple, and sextuple binding events are shown in Fig. 2, *a* and *c*. According to the hexameric structure of hemichannels, angles of 60° between adjacent subunits should be observed. Angle analysis for the double Fab-HA binding in the homomeric Cx hemichannels showed three peaks centered at 58°, 122°, and 169° (Fig. 2*b*) near to expected angles multiple of 60°. As shown in Table 1, only single and double nonrelated Fab-V5 antibody fragment (Fab-V5) bindings were observed, corresponding to 6- and 12-fold smaller events than those using the Fab-HA decoration, indicating that nonspecific binding events are not significant in this study.

**Table 1:** Quantification of Cx hemichannel/Fab-epitope complexes under experimental and simulated conditions Number of Cx particles and binding percentages are indicated. Simulated data based on binomial distribution was obtained at α = 0.19. (—) represents the absence of sextuple Fab-Cx26 binding because HA-affinity purification was carried out on the Cx30-HA/Cx26 heteromer.

Hemichannels	Antibodies	Nonbinding	Single binding	Double binding	Triple binding	Quadruple binding	Quintuple binding	Sextuple binding
Cx30-HA/Cx26 experimental	Fab-Cx26	400 (67.7%)	168 (28.4%)	19 (3.2%)	4 (0.7%)	0 (0%)	0 (0%)	— (—)
Cx26-HA/Cx30 experimental	Fab-HA	260 (44.1%)	236 (40.1%)	79 (13.4%)	14 (2.4%)	0 (0%)	0 (0%)	0 (0%)
Cx30-HA/Cx26 experimental	Fab-HA	279 (43.8%)	272 (42.7%)	75 (11.8%)	11 (1.7%)	0 (0%)	0 (0%)	0 (0%)
Cx26-HA/Cx30 or Cx30-HA/Cx26 theoretical	Fab-HA	(54.2%)	(35.2%)	(9.2%)	(1.3%)	(0.1%)	(0%)	(0%)
Cx26-HA experimental	Fab-V5	134 (90.0%)	13 (8.7%)	2 (1.3%)	0 (0%)	0 (0%)	0 (0%)	0 (0%)
Cx26-HA experimental	Fab-HA	148 (28.2%)	260 (49.8%)	81 (15.5%)	29 (5.5%)	4 (0.8%)	0 (0%)	1 (0.2%)
Cx26-HA theoretical	Fab-HA	(28.2%)	(39.8%)	(23.3%)	(7.3%)	(1.3%)	(0.1%)	(0%)

**Figure 3:**
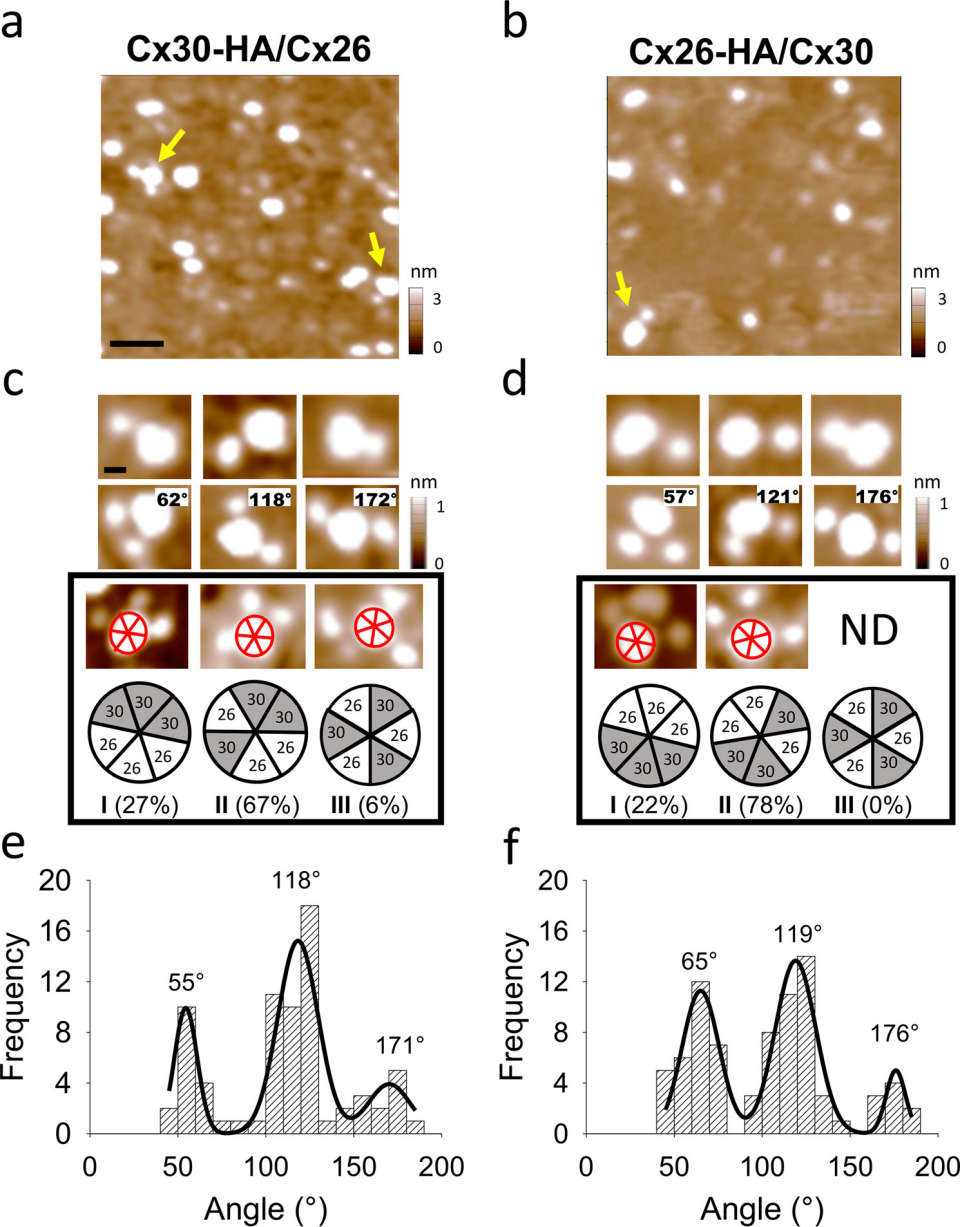
**Fab-HA decoration of heteromeric Cx30-HA/Cx26 and Cx26-HA/Cx30 hemichannels.***a* and *b*, AFM imaging of co-incubated Fab-HA and Cx30-HA/Cx26 or Cx26-HA/Cx30 hemichannels. *Yellow arrows* show single and double binding (*scale bar*, 50 nm). *c* and *d*, images show different binding numbers (1, 2, and 3) of Fab-HA and different angles of two Fab-HA (indicated in the *right corner*) bound to the hemichannels (*scale bar*, 20 nm). Abundance of the 3Cx26:3Cx30 heteromer *arrangements* (*I*, *II*, *III*) is indicated. *ND*, not detected. *e* and *f*, frequency distribution of angles from double binding.

Heteromeric hemichannels were incubated with Fab-HA and imaged by AFM. In low magnification images (Fig. 3, *a* and *b*), three populations of particles were observed, small ones corresponding to Fab-HA, larger ones corresponding to heteromeric hemichannels and complexes corresponding to interacting Fab-HA–Cx hemichannel. Most of the Cx hemichannels (Cx26-HA/Cx30 and Cx30-HA/Cx26) were containing up to three Fab-HA bound and the proportion of bindings is similar between both complexes (Table 1). Fig. 3, *c* and *d* shows a gallery of AFM images with one, two, and three Fab-HA bound to either Cx26-HA/Cx30 or Cx30-HA/Cx26. These indicate a data consistency on the stoichiometry between both heteromeric complexes independent of what subunit (Cx26 or Cx30) expressed the HA tag and the cellular machinery involved in each transfection. The heteromeric Cx hemichannels also seem to follow relatively well the binomial binding distribution. The absence of four, five, or six Fab-HA binding events should be theoretically expected in the heteromers, in contrast to the larger theoretical decoration in the homomer (10-fold more binding events than heteromer), which is consistent with the corresponding experimental binding pattern (Table 1). Theoretical calculations by simulating the abundances of heteromeric Cx hemichannels with different stoichiometries also predicted that a larger population of 3Cx26:3Cx30 fits better to the Fab-HA experimental binding pattern (lowest mean squared error, MSE) (Equation 5, see “Experimental procedures”). Thus, theoretical and experimental data strongly suggest that purified heteromeric Cx26/Cx30 hemichannels have predominantly a stoichiometry of 3:3.

### Subunit arrangement of the Cx26/Cx30 hemichannels

To resolve the subunit arrangement within the heteromeric Cx hemichannel (see all possible subunit arrangements and stoichiometries in Fig. S2), angles between two Fab-HA were analyzed. Three different angles were observed and are summarized in the histograms shown in Fig. 3, *e* and *f*; the peaks were centered at 55°, 118°, and 171° for the Cx30-HA/Cx26 and 65°, 119°, and 176° for the Cx26-HA/Cx30. These data suggest that Fab-HA bound subunits can be either adjacent (expected angle 60°) or separated by another subunit (expected angle 120°) or separated by two subunits (expected angle 180°). The proportion of the angles is around 40%, 40 and 20% for 60°, 120°, and 180°, respectively, which fulfil very well a random ensemble of Cx monomers calculated by the law of total probabilities (LTP). LTP defines that the probability of an unknown arrangement composed by *n* subunits can be determined using the known probabilities of arrangements from *n-1* subunits, *i.e.* if a Cx30 subunit is present then the following Cx30 at 60° separation has 2 out of 5 (40%) positions available in the hexamer (Equation 6, see “Experimental procedures”).

In the triple binding analysis, three different subunit arrangements were observed (Fig. 3, *c* and *d*), however, the relative abundance of each one was different. The symmetric subunit arrangement, corresponding to Cx26-Cx30 interfaces and angles only at 120° were the least abundant with only 6 and 0% of total particles analyzed for Cx30-HA/Cx26 and Cx26-HA/Cx30, respectively (*arrangement III* in Fig. 3, *c* and *d*). The subunit arrangement with three identical adjacent subunits, forming angles at 60° and 120°, represents 27 and 22% of total particles analyzed for Cx30-HA/Cx26 and Cx26-HA/Cx30, respectively (*arrangement I* in Fig. 3, *c* and *d*). Finally, the subunit arrangement and most abundant with 67 and 78% of total particles analyzed for Cx30-HA/Cx26 and Cx26-HA/Cx30 respectively, has angles at 60°, 120°, and 180°, corresponding to two equal adjacent subunits and a third subunit separated by a different one (*arrangement II* in Fig. 3, *c* and *d*). Note that this latter subunit arrangement is the only one containing the three different angles observed after analyzing double binding events. Interestingly, by calculating again a random ensemble of Cx monomers via LTP now to the proportion of angles in triple binding events in the heteromeric Cx26/Cx30 hemichannels, our experimental data match relatively well, because *arrangement III* is the less abundant (10%), followed by *arrangement I* (30%), and the most abundant *arrangement II* (60%) (see details under “Experimental procedures”).

**Figure 4:**
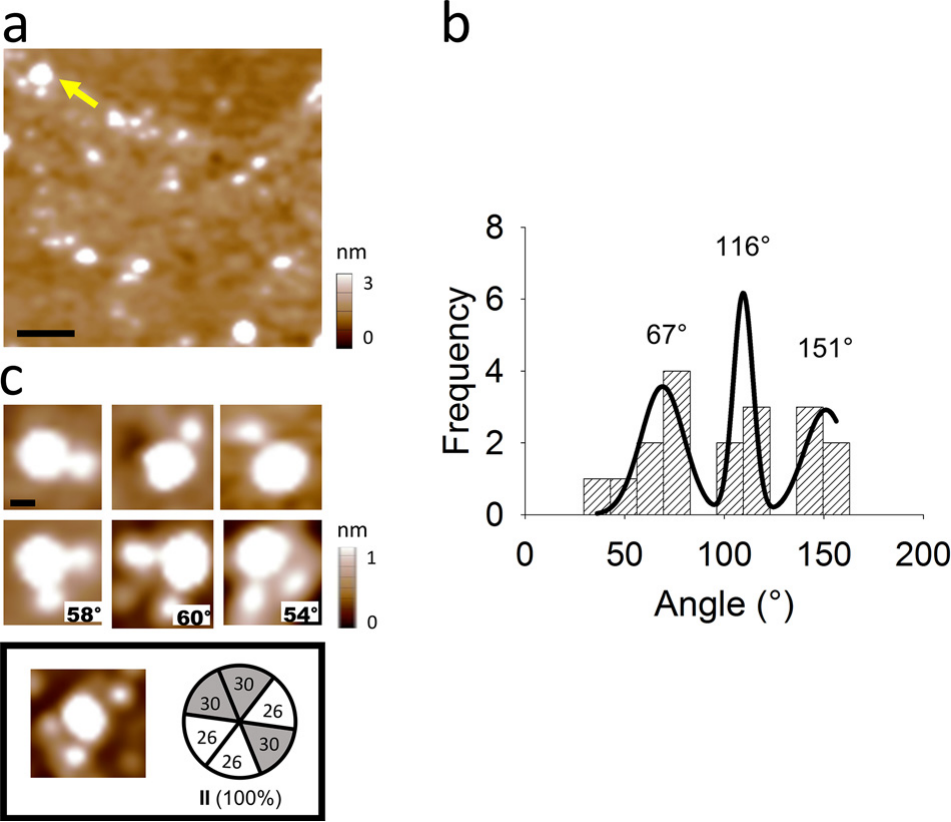
**Heteromeric Cx30-HA/Cx26 hemichannels decorated with Fab-Cx26.***a*, AFM imaging of co-incubated Cx30-HA/Cx26 hemichannels and Fab-Cx26. *Yellow arrow* shows double Fab-Cx26 binding (*scale bar*, 50 nm). *b*, frequency distribution of angles from double binding. *c*, images show different binding numbers (1, 2, and 3) of Fab-Cx26 and different angles of two Fab-Cx26 (indicated in the *right corner*) bound to the hemichannels (*scale bar*, 20 nm). Abundance of the 3Cx26:3Cx30 heteromer *arrangement II* is indicated.

Finally, heteromeric Cx26/Cx30-HA hemichannel was analyzed by Fab-Cx26 antibody fragment (Fab-Cx26). AFM imaging showed populations of Cx heteromers bound with one, two, and three Fab-Cx26 (Fig. 4, *a–c*), similar to the Fab-HA decoration. Although the number of triple Fab-Cx26 bindings was lower than using Fab-HA (Table 1), it is relevant to highlight that 100% of these events had the same subunit arrangement (Fig. 4*c*) observed with the preponderant Fab-HA decoration (*arrangement II* in Fig. 3, *c* and *d*).

### Stoichiometry and arrangement of the ringlike structures of heteromeric Cx26/Cx30 hemichannels

**Figure 5:**
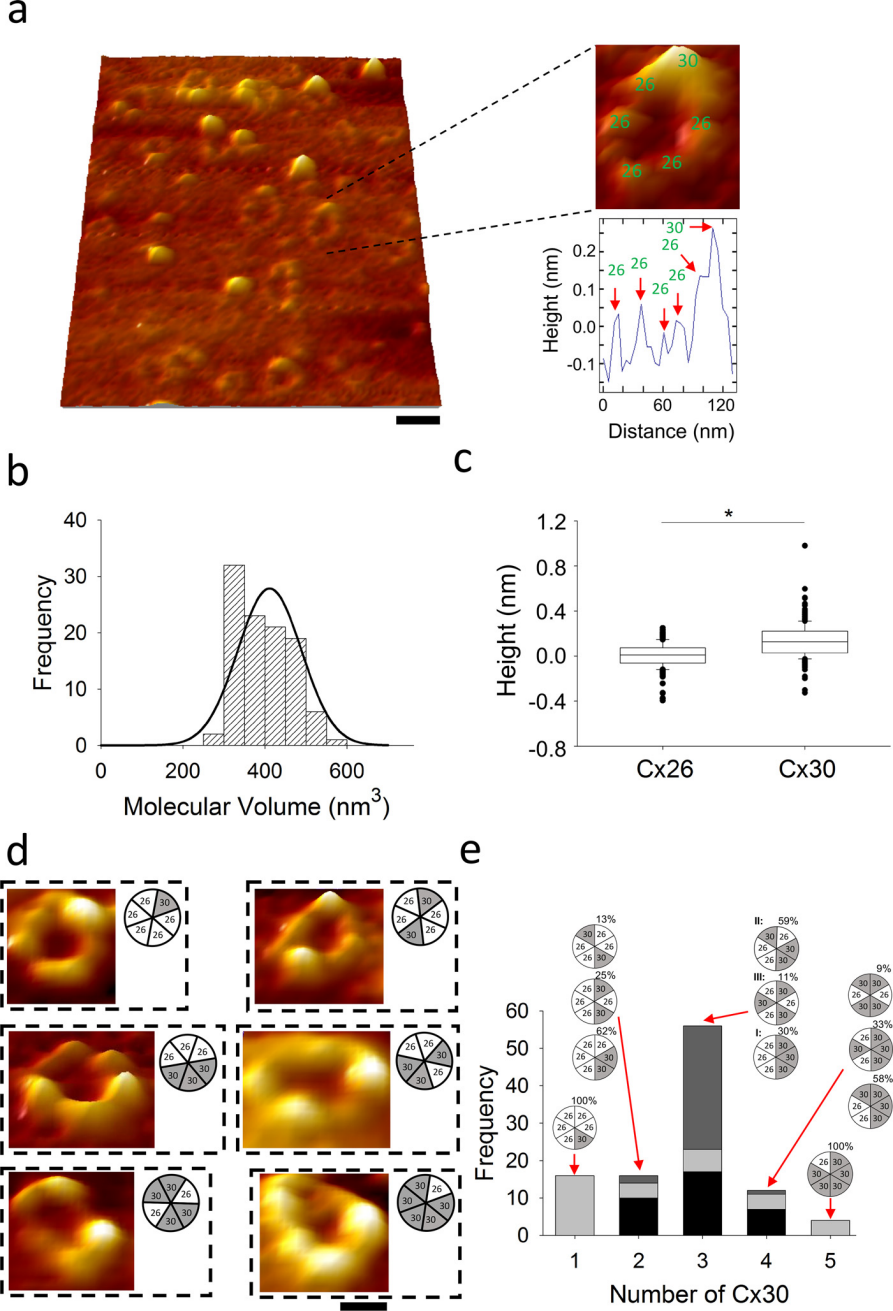
**AFM imaging of ring-like structures of heteromeric Cx30-HA/Cx26 hemichannels.***a*, AFM imaging of ringlike structures of Cx30-HA/Cx26 hemichannels adsorbed into mica. *Left*, three-dimensional images of adsorbed hemichannels (*scale bar*, 50 nm). *Right*, zoomed three-dimensional image of an individual heteromeric hemichannel indicating six peaks with the corresponding section analysis. Numbers 26 and 30 represent Cx isoform. *b*, frequency distribution of molecular volumes of ringlike structures. *Black curve* indicates fitted Gaussian function. *c*, box-plot of height of Cx26 and Cx30. Asterisk (*) represents P < 0.05, unpaired *t* test. *d*, representative three-dimensional images of ringlike structures of several Cx30-HA/Cx26 hemichannel stoichiometries and arrangements (*scale bar*, 30 nm). *e*, frequency distribution of stoichiometries and arrangements of heteromeric Cx30-HA/Cx26 hemichannels in function of the number of Cx30-HA present. *Arrows* indicate the arrangement and abundance (%) of the specified stoichiometry (*n* = 16, 16, 56, 12, and 4 for hemichannels containing 1, 2, 3, 4, and 5 Cx30-HA, respectively). Note that for the 3Cx30:3Cx26 stoichiometry, nomenclature of *arrangements I*, *II*, and *III* is also included.

The physical chemistry underlying protein adsorption onto mica is a complex process, and it is possible to observe partially denatured macromolecules, which makes hemichannels flatter and less compact so the pore and the subunit edges can be better resolved within ringlike structural shape (Fig. 5*a*). Via a section analysis of these structures, a 6-peak pattern can be recorded (Fig. 5*a*, *right*), corresponding to six individual spheres resulting in an overall molecular volume of 411 ± 11 nm^3^ (*n* = 104) (Fig. 5*b*), a peak value close to that of single Cx30-HA/Cx26 hemichannels (Fig. 1*e*) (although statistically smaller via unpaired *t* test, *p* = 0.03). Each Cx isoform should have characteristic dimensions such as height according to their different molecular weights. The peak height distribution has shown two populations significantly different (Fig. 5*c*) (*p* < 0.05, unpaired *t* test) which could be assigned to Cx26 and Cx30 subunits. Therefore, the subunit stoichiometry and arrangement can be determined independent of the Fab binding characterization.

It was experimentally observed a polydisperse distribution of stoichiometries for the heteromeric hemichannels, corresponding to 5Cx26:1Cx30 (15% abundance), 4Cx26:2Cx30 (15% abundance), 3Cx26:3Cx30 (54% abundance), 2Cx26:4Cx30 (12% abundance), and 1Cx26:5Cx30 (4% abundance) (Fig. 5, *d* and *e*) which resembles a binomial distribution. In addition, a variety of arrangements were observed within each stoichiometry which could not be identified by the Fab binding approach. The predominant stoichiometry for the ringlike structures of heteromeric hemichannels is represented by the 3Cx26:3Cx30 composition; importantly, the abundance for its *arrangement I* (30%), *II* (59%), and *III* (11%) is similar to those detected by the Fab binding approach (Fig. 3).

Taken together, our results herein suggest that the most abundant stoichiometry for the heteromeric Cx26/Cx30 hemichannel is 3:3 with the major subunit arrangement Cx26-Cx26-Cx30-Cx26-Cx30-Cx30.

## Discussion

AFM imaging of heteromeric membrane proteins complexed to anti-epitope antibodies has been a validated experimental technique to resolve the molecular architecture of a considerable number of ion channels, transporters, and receptors (11, 12, 13, 14, 15, 16, 17, 18, 19, 20, 21, 22, 23, 24, 25), which can present either a fixed or a variable subunit arrangement. Here, we showed that heteromeric Cx26-HA/Cx30 and Cx26/Cx30-HA hemichannels, expressed and purified after transfection of equally subunit cDNA amounts from bicistronic vectors, have a preponderant subunit stoichiometry of 3Cx26:3Cx30 and a preferential subunit arrangement of Cx26-Cx26-Cx30-Cx26-Cx30-Cx30 (*arrangement II* in Fig. 3, *c* and *d*).

Heteromeric Cx26-HA/Cx30 and Cx26/Cx30-HA hemichannels presented a tendency for binomial Fab-HA binding distribution with similar binding events between each other suggesting that (i) the Fab-HA affinity for the tag position (Cx26-HA or Cx30-HA) and (ii) the cell machinery during transfection with different constructs, did not affect the oligomerization pattern of hemichannels. Although the Fab-HA binding efficiency cannot be determined precisely, we can assume that it should be similar between homomeric and heteromeric forms. In consequence, at a Fab-HA binding efficiency of 0.19, both homomeric and heteromeric forms present a similar tendency for the Fab-HA binomial binding events (similar MSE values, see “Experimental procedures”). Furthermore, if we increase the abundance of 3Cx26:3Cx30 stoichiometry within the binomial distribution, the Fab-HA binding pattern fits better to the experimental data (lowest MSE value, see “Experimental procedures”). Taken together, this supports the idea that four, five, or six Fab-HA bindings into the Cx heteromers, would not represent the majority population of stoichiometries. Using an additional experimental strategy aimed to visualize ringlike structures of heteromeric hemichannels, without the assistance of Fab binding events, we have been able to confirm these findings. Similarly, the 3Cx26:3Cx30 stoichiometry was predominant and three 3Cx26/3Cx30 arrangements with different abundances were observed. Furthermore, additional stoichiometries and arrangements were observed in our purified heteromeric hemichannels supporting the notion of structurally heterogeneous population of heteromeric hemichannels (30). This is consistent with a variety of permeabilities observed in reconstituted heteromeric Cx26/Cx32 hemichannels (9). Interestingly, our 3Cx26/3Cx30 subunit arrangements can be theoretically calculated via LTP approach, which demonstrates that the heterooligomerization process could occur randomly via a monomeric assembly, where subunit interfacial features would be conserved. Coincidently, the proposed assembly mechanism of β type Cxs, such as Cx26 and Cx30 hemichannels, should happen via unstable monomers in the endoplasmic reticulum (31, 32, 33, 34). In addition, via molecular dynamics simulations of *in silico* built models for Cx46/Cx50 heteromers, in this case α type Cxs, results in highly similar interfacial interactions (10). Further structural studies to confirm the stoichiometry of heteromeric Cx hemichannels might be done via complementary methods such as native MS, as we have already shown on several membrane protein complexes (35, 36, 37, 38, 39), although a considerable increase in protein concentration and stability should be required prior to undertake this new task. Nevertheless, that approach is not able to provide the subunit arrangement, which can be effectively stated herein via AFM imaging. Although protein stability and/or denaturation may play a role on the purified Cx26/Cx30 heteromeric hemichannels, it is unlikely that subunit stoichiometry and/or arrangement of full intact complexes could change during this isolation process. However, we cannot ensure that these structural features correspond to those present in native cells, and additional challenging strategies should be applied to visualize them directly in a cellular context.

Cx hemichannels are selectively permeable to biological molecules including cAMP, cGMP, and inositol phosphates (30). Nevertheless, different selectivity to molecules for homomeric and heteromeric hemichannels has been reported. For example, whereas Cx26 and Cx32 homomeric hemichannels were shown fully permeable to different inositol triphosphates isomers, the corresponding Cx26/Cx32 heteromeric hemichannels show selectivity to some of the isomers. In this particular case, heteromeric channels display higher selectivity than homomeric channels for these molecules. This may be biologically important given that Cx26 and Cx32 can co-exist forming heteromeric channels in some tissues (*i.e.* liver), but not others (*i.e.* skin and Schwann cells) offering distinctive selectivity to cell signaling molecules depending on the cell type and the connexins that are expressed. Similar structural arrangement and functional properties are expected for Cx26 and Cx30 depending on their tissue expression pattern. It remains to be answered, however, what are the selective and permeability properties of each Cx26/Cx30 heteromeric arrangement identified in this work. Up to now, it was technically unfeasible to isolate and assess properties for a single hemichannel at the same time knowing the structural arrangement and stoichiometry of the different subunits. Without this information, the biological implication of individual population of heteromeric hemichannels still remains to be further dissected.

In summary, we showed for the first time the molecular architecture of purified heteromeric hemichannels formed by Cx26/Cx30, which has not been available by other methodological approaches. Our data strongly suggest the presence of Cx26/Cx30 hemichannels in a polydisperse manner with a preponderant 3:3 stoichiometry and subunit arrangement of Cx26-Cx26-Cx30-Cx26-Cx30-Cx30. This provides novel insights into the mechanism of heteromeric Cx oligomerization and paves the way to functional analysis of transport through these hemichannels.

## Experimental procedures

### Expression of tagged connexin hemichannels in HeLa cells

Bidirectional tetracycline-responsive expression vectors (Clontech), containing one or two multiple cloning sites, were used to express homomeric or heteromeric Cx hemichannels in HeLa cells, which have virtually no endogenous Cx expression (40). For expression of homomeric channels, human Cx26 or Cx30 coding sequences were subcloned into one cloning site in frame with a sequence coding for a carboxyl-terminal tag consisting of a thrombin cleavage site followed by a HA epitope (not His-Ala) and (His-Asn)6, *i.e.* a HA(HN)6 tag. When both cloning sites contained different connexin coding sequences, only one connexin was tagged. “Tet-On” cell lines were maintained in 200 μg/ml hygromycin and 100 μg/ml G418. Channels are termed as Cx26Tag (Cx26-HA) or Cx30Tag (Cx30-HA) when homomeric and as Cx26-HA/Cx30 or Cx26/Cx30-HA when heteromeric.

### Purification of tagged connexin hemichannels

Cells at 35% confluence in four 500-cm^2^ dishes were induced with 1g/ml doxycycline for 48 h, during which time the expressed Cx(s) formed functional gap junctions as the cells became confluent. Cells were solubilized in 50 mm NaH_2_PO_4_, 5 mm NaCl, 5 mm EDTA, 5 mm EGTA, 80 mm OG (octyl-β-glucoside), 1 mm β-mercaptoethanol, 0.5 mm diisopropyl fluorophosphate (Calbiochem), 0.75 mg/ml azolectin, pH 7.5, for 2 h at 4°C with rocking. Solubilization of gap junctions with OG yields hemichannels (9).

The supernatant (100,000 × *g* for 30 min at 4°C) was incubated with 0.25 ml of agarose-immobilized anti-HA mouse IgG overnight at 4°C with shaking. The antibody matrix was collected by centrifugation at 700 × *g* for 1 min at 4°C and washed in a fritted column with 20 ml of 10 mm PBS, 1M NaCl, 80 mm OG, 1 mg/ml azolectin, pH 7.4, followed by 20 ml of the same solution containing only 138 mm NaCl. Hemichannels were eluted with 5 mm CH_3_COOHNa, 0.5 m NaCl, 10 mm KCl, 1 mm EDTA, 80 mm OG, pH 4.0, and 0.6-ml fractions were collected into 0.05 ml of 1 m NaHCO_3_, 10 mm KCl, 80 mm OG, pH 9.0. The final pH was ∼7.4.

### Western blot analysis

Purified samples were analyzed by SDS-PAGE, followed by immunoblotting, using mouse monoclonal anti-HA (Thermo Fisher) and rabbit polyclonal anti-Cx26 antibody (Thermo Fisher cat. no. 51-2800), followed by horseradish peroxidase–conjugated goat anti-mouse or anti-rabbit antibody (Thermo Fisher). Inmunoreactive bands were visualized using enhanced chemiluminescence.

### Tandem MS of heteromeric hemichannels

Briefly, protein sample of heteromeric Cx30-HA/Cx26 hemichannels were precipitated with ethanol and the pellet was dissolved in 10 μl 1% (m/v) RapiGest SF Surfactant (Waters). Disulfide bridges were reduced with 50 mm DTT and reduced cysteines were alkylated with 100 mm iodoacetamide. Proteins were digested with 0.5 μg trypsin (Promega) at 37°C overnight. RapiGest was removed by centrifugation after addition of 5% (v/v) TFA. Tryptic peptides were separated by nano-flow reversed-phase liquid chromatography using a DionexUltiMate 3000 RSLC nano System (Thermo Scientific) and directly eluted into an LTQ-Orbitrap XL hybrid mass spectrometer (Thermo Scientific). MS conditions were spray voltage of 1.8 kV, capillary temperature of 180°C, normalized collision energy of 35% at an activation of *q* = 0.25 with activation time of 30 ms. MS spectra were acquired in the Orbitrap (*m*/*z* 300–2000) at a resolution of 30,000 at *m*/*z* 400. The five most intense ions were selected for collision-induced dissociation MS/MS fragmentation in the linear ion trap. Previously detected ions were excluded for 30 s. Singly charged ions and unrecognized charge states were excluded for fragmentation. Mass spectra were internally calibrated using the lock mass *m*/*z* 445.120025 (41).

Raw data were searched against the NCBInr database using the search engine Mascot v2.3.02. Mass accuracy filters were 10 ppm (precursor ions) and 0.5 Da (fragment ions). Two missed cleavage sites per tryptic peptide were allowed. Variable modifications were carbamidomethylation (cysteines) and oxidation (methionine).

### AFM imaging of purified connexin hemichannels

Purified protein samples were diluted and 30 μl of the sample was allowed to adsorb to freshly cleaved mica disks. After a 5-min incubation, the sample was washed with Milli-Q water and dried under nitrogen. Imaging was performed with an MFP-3D AFM (Asylum Research Instrument). Samples were imaged in air, using tapping mode. The silicon cantilevers used had a drive frequency of ∼300 kHz and a specified spring constant of 42 newtons/meter (Asylum Research). The applied imaging force was kept as low as possible (As/A0 ∼0.85), where As and A0 correspond to the amplitude set point and free amplitude, respectively. The heights and half-height radii were measured from cross-section for each particle; these data were used to calculate molecular volumes according to

(Eq. 1) $$Vm=\left(\pi h/6\right)\left(3r^{2}+h^{2}\right)$$

where *h* is the particle height and *r* is the radius (29). This equation assumes that the adsorbed particles adopt the form of a spherical cap. Particle volumes were calculated using a homemade script combining Igor pro and the MFP-3D software. Because ringlike structures of connexin hemichannels are relatively flat, their molecular volumes were calculated similarly, considering two cross-section analyses with their averaged height and averaged radius between the maximum and minimum values. No differences in their molecular volumes were found comparing this method with grain analysis of segmented structures (*p* > 0.05, unpaired *t* test).

Molecular volume based on molecular mass was calculated using

(Eq. 2) $$Vc=\left(M_{0}/N_{0}\right)\left(V1+dV2\right)$$

where M_0_ is the molecular mass, N_0_ is Avogadro's number, *V*1 and *V*2 are the partial specific volumes of particle (0.74 cm^3^/g) and water (1 cm^3^/g), respectively, and *d* is the extent of protein hydration (taken as 0.4 g of water/g of protein).

### AFM imaging of connexin hemichannels bound to Fab fragments

Purified proteins were mixed with Fab antibody fragments derived from anti-HA or anti-Cx26 or anti-V5 antibody (Fab fragment preparation was carried out with the Pierce^TM^ Fab Preparation Kit) in a ratio 50:1 and incubated overnight with constant shaking. Next day, Cx-Fab fragment complexes were concentrated and separated from free Fab-HA fragments using Amicon® Ultra 100K. The complex was adsorbed into the mica as described above.

The criteria that must be met to consider binding events were (i) bound Fab fragments have a molecular volume between 40 and 150 nm^3^, (ii) a cross-section was drawn through the junction between the peripheral and the central particle (Fab fragment and Cx hemichannel, respectively), and the height of the lowest point between the two particles has to be ≥ 0.2 nm, (iii) if a particle resembles an ellipsoid rather than a circle, an averaged radius between the maximum and minimum values is considered in Equation 1, and (iv) any particle is rejected if its length is greater than twice its width (42).

### Binomial distribution simulation of the Cx hemichannel Fab-HA binding

To calculate the binding efficiency between Fab-HA and Cx hemichannel based on binomial distribution (43), the probability of finding 0, 1, 2, 3, 4, 5, or 6 Fab bound are given by

(Eq. 3) PiFab-HA=6iαi(1-α)6-i

where *i* and α represent the number of Fab-HA bound and binding efficiency, respectively.

The binomial distribution could also determine the abundance of the various stoichiometries of the heteromeric Cx26/Cx30 hemichannels, then for example 1000 Cx26/Cx30 hemichannels with stoichiometries 6:0, 5:1, 4:2, 3:3, 2:4, 1:5, and 0:6 should have abundances of 15.6, 93.7, 234.4, 312.5, 234.5, 93.7, and 15.6, respectively. However, as the HA-tag is present only in one subunit *i.e.* Cx26-HA, then the stoichiometry 0:6 was not analyzed because homomeric Cx30 hexamer cannot be pulled down under the purification conditions. The corrected binomial distribution can be derived as

(Eq. 4) 1000×P6:i=6i×0,561-P(0:6)

where the corrected abundances for stoichiometries 6:0, 5:1, 4:2, 3:3, 2:4, and 1:5 are 15.8, 95.2, 238.1, 317.6, 238.1, and 95.2, respectively.

The MSE value, between experimental and theoretical binomial binding, was calculated as

(Eq. 5) $$MSE=\frac{\sum_{i=0}^{6}{\left(P_{experimentaliFab-HA}-P_{theoreticaliFab-HA}\right)}^{2}}{7}$$

For the MSE analysis, Cx26-HA homomer as well as the averaged Cx26-HA/Cx30 and Cx26/Cx30-HA heteromers were considered.

Once incubated with Fab-HA, the selection of unbound hexameric Cx hemichannels based on AFM imaging could be overestimated because of protein adsorption in the incorrect orientation for analysis, *i.e.* Fab-HA bound on top of the adsorbed hemichannel. Therefore, the effective number of unbound Cx hexamers (P*_experimental 0_*_Fab_) was normalized by the corresponding P*_theoretical 0_*_Fab_ using the homomeric Cx hemichannel simulation. A binding efficiency of 0.19 was chosen because at that point both homomeric and heteromeric forms present similar MSE values (27.4 and 26.4, respectively). Note that increasing the abundance of the 3Cx26:3Cx30 stoichiometry within the heteromers over the 4Cx26:2Cx30 and 2Cx26:4Cx30 stoichiometries, *i.e.* (60:20:20) and (80:10:10), gives smaller MSE values of 22.2 and 20.9, respectively.

### LTP for random Cx subunit arrangement within the heteromeric 3Cx26/3Cx30 hemichannel

Let's say we have an array like this for the Cx26-HA hemichannel including one Cx30 subunit:



After this, we want to put the second Cx30 subunit, for which there are three different possibilities:



By using LTP (44),

(Eq. 6) $$P\left(A\right)=\sum_{1}^{n}P\left(A|X_{n}\right)P(X_{n})$$

P(A|X): Probability of arrangement A given arrangement X. P(X): Probability of arrangement X

Given arrangement X, it is clear that the probability of getting arrangements A, B, and C corresponds to 0.4, 0.4, and 0.2, respectively.

Now, to calculate the probabilities of arrangements where three subunits for each Cx are present, we need to count the number of events for each structure and the total number of events.



For example, the probability of having the arrangement II given A (*i.e.* P(II|A)) is 0.5, because there are four spots to add the next subunit and two of them gives us II. The probability of having the arrangement II given B is 0.5, because there are two out of four spots that give us the structure II. Finally, the probability of having the arrangement II given C is 1, because there are four spots that give us the structure II, and now using the LTP, the probability of having the arrangement II is P(II)= P(II|A) ×P(A)+ P(II|B) ×P(B)+ P(II|C) ×P(C)=0.5 × 0.4 + 0.5 × 0.4 + 1 × 0.2 = 0.6.

Similarly, by doing the same analyses for arrangements I and III, the probabilities are 0.3 and 0.1, respectively.

## Data availability

All data are contained within the manuscript. Additionally, the MS proteomics data have been deposited to the ProteomeXchange Consortium via the PRIDE (45) partner repository with the dataset identifier PXD020633.

10.13039/501100002850MINEDUC | CONICYT | Fondo Nacional de Desarrollo Científico y Tecnológico (FONDECYT) (3160568) to Pamela A. Naulin
Millennium Science Initiative (P10-035F) to Nelson P. Barrera
10.13039/100004440Wellcome Trust (Wellcome) (088150/Z/09/Z) to Nelson P. Barrera
NIH (RO1-GM099490) to Jorge E. Contreras
NIH (RO1-GM101950) to Jorge E. Contreras
Fondequip (EQM150102) to Nelson P. Barrera
Fondequip (EQM170172) to Nelson P. Barrera
