# Supplementary material for: Polydisperse molecular architecture of connexin 26/30 heteromeric hemichannels revealed by atomic force microscopy imaging
Source: J Biol Chem. 2021 Jan 13;295(49):16499–509. doi: 10.1074/jbc.RA119.012128 (PMC7864052; doi:10.1074/jbc.RA119.012128)
Supplement: Supplementary file 1 [file mmc1.pdf]

SUPPORTING INFORMATION

Polydisperse molecular architecture of connexin 26/30 heteromeric hemichannels revealed by atomic force microscopy imaging

Pamela A. Naulin[1], Benjamin Lozano[1], Christian Fuentes[1], Yu Liu[2], Carla Schmidt[3,#], Jorge E. Contreras[2], and Nelson P. Barrera[1,*]

From the [1] Department of Physiology, Faculty of Biological Sciences, Pontificia Universidad Católica de Chile, Alameda 340, 8331150, Santiago, Chile; [2] Department of Pharmacology, Physiology and Neuroscience, New Jersey Medical School, Rutgers University, Newark, NJ 07103; [3] Department of Chemistry, University of Oxford, Oxford, OX1 3QZ, United Kingdom

Running title: *Molecular architecture of connexin heteromeric hemichannels*

[#] Present Address: Interdisciplinary Research Center HALOmem, Charles Tanford Protein Center, Institute for Biochemistry and Biotechnology, Martin Luther University Halle-Wittenberg, 06120 Halle, Germany

[*] To whom correspondence should be addressed: Nelson P. Barrera: Department of Physiology, Faculty of Biological Sciences, Pontificia Universidad Católica de Chile, Alameda 340, 8331150, Santiago, Chile; nbarrera@bio.puc.cl; Tel. (56-2) 2354-2872.

Supporting information contents:

**Figure S1.**	Molecular volumes obtained of homomeric Cx30-HA hemichannels, anti-HA antibody and Fab-HA from AFM imaging.
**Figure S2.**	Schematic representation of the possible stoichiometry and subunit arrangement of the heteromeric Cx26/Cx30 hemichannels.
**Table S1.**	MS database search result for Cx26 and Cx30.
**Table S2.**	Theoretical molecular volumes, considering all possible stoichiometries, of both heteromeric Cx26-HA/Cx30 and Cx30-HA/Cx26 hemichannels.

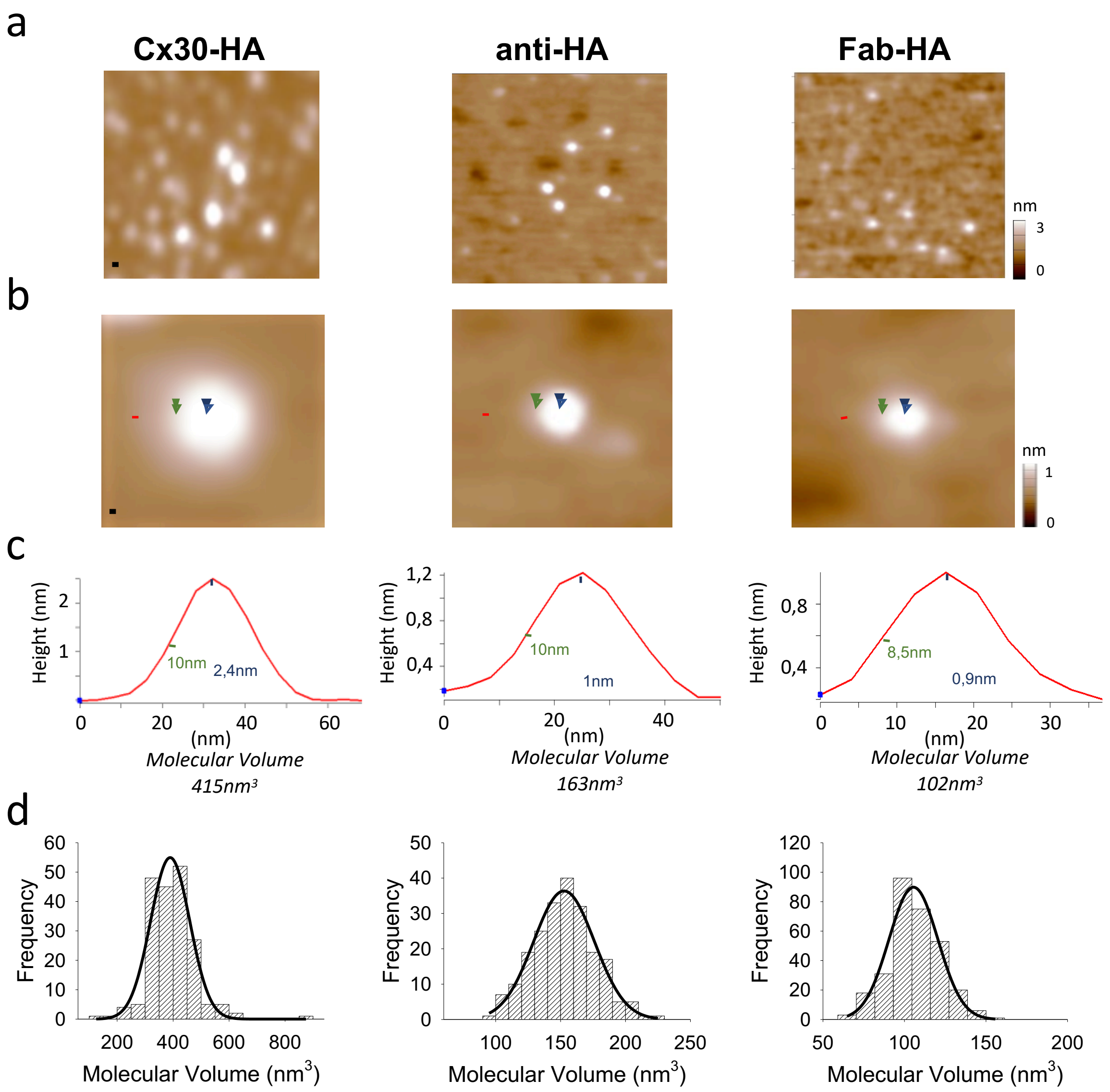

Figure S1. Molecular volumes obtained of homomeric Cx30-HA hemichannels, anti-HA antibody and Fab-HA from AFM imaging. *a*, Low-magnification image (scale bar 50nm). *b*, High-magnification image of single proteins. Sections through particles are shown as red lines including two points, height and radius at half height (blue and green arrows respectively) (scale bar 20nm). *c*, Particle height analysis of the indicated section. *d*, Frequency distribution of molecular volumes. Black curves indicate fitted Gaussian functions.

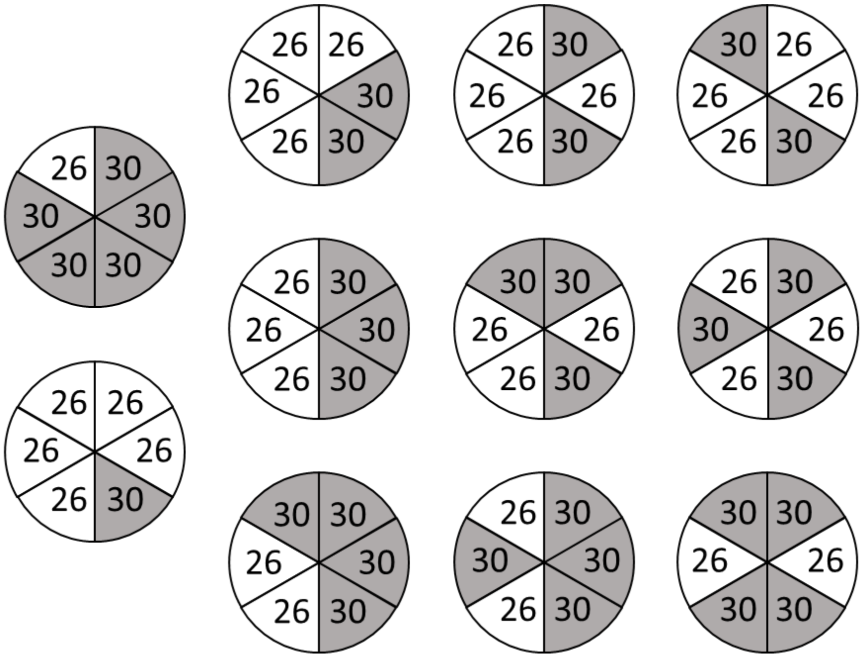

Figure S2. Schematic representation of the possible stoichiometry and subunit arrangement of the heteromeric Cx26/Cx30 hemichannels. 26 and 30 represent Cx26 and Cx30 respectively.

m/z exp.	MW exp.	charge state	MW theor.	delta MW	MC	MPS	peptide sequence	modification
				Cx26				
321.5395	961.5967	3	961.596	0.0006	2	26.06	KFIKGEIK	n/a
341.8942	1022.6608	3	1022.66	0.0007	1	37.12	SKKPVLVPR	n/a
552.3013	1102.588	2	1102.587	0.0011	1	10.29	DIEEIKTQK	n/a
532.2816	1593.823	3	1593.825	-0.002	2	35.39	SEFKDIEEIKTQK	n/a
532.2826	1593.826	3	1593.825	0.001	2	43.29	SEFKDIEEIKTQK	n/a
816.9247	1631.8348	2	1631.8341	0.0007	0	62.78	MDWGTLQTILGGVNK	n/a
				Cx30				
366.6925	731.3704	2	731.3701	0.0003	0	17.86	DIEDIK	n/a
366.6926	731.3706	2	731.3701	0.0005	0	14.19	DIEDIK	n/a
465.7478	929.481	2	929.4831	-0.0021	0	12.68	NHPNHALK	n/a
310.8346	929.482	3	929.4831	-0.0012	0	29.33	NHPNHALK	n/a
465.7486	929.4826	2	929.4831	-0.0005	0	10.23	NHPNHALK	n/a
310.8352	929.4838	3	929.4831	0.0006	0	33.66	NHPNHALK	n/a
310.8357	929.4853	3	929.4831	0.0021	0	25.88	NHPNHALK	n/a
319.4205	1273.6529	4	1273.6527	0.0002	1	13.65	NHPNHALKESK	n/a
455.5732	1363.6978	3	1363.6983	-0.0005	2	55.57	NDFKDIEDIKK	n/a
682.8568	1363.699	2	1363.6983	0.0007	2	26.29	NDFKDIEDIKK	n/a
455.5737	1363.6993	3	1363.6983	0.001	2	45.08	NDFKDIEDIKK	n/a
496.267	1485.7792	3	1485.78	-0.0009	1	12.04	AQTQKNHPNHALK	n/a
559.2802	1674.8188	3	1674.8188	-0.0001	0	17.36	MDWGTLHTFIGGVNK	n/a
458.4938	1829.9461	4	1829.9496	-0.0035	2	14.35	AQTQKNHPNHALKESK	n/a

Table S1. MS database search result for Cx26 and Cx30. For all identified peptides, the experimentally determined m/z (m/z exp.), the experimentally determined molecular weight (MW exp.), the charge state, the theoretical molecular weight (MW theor.), the mass difference (delta MW), the number of missed cleavage sites (MC), the Mascot peptide score (MPS), the peptide sequence and identified modifications are listed.

Connexin type	Subunit ratio	Theoretical volume of hemichannel (nm^3)	Theoretical volume of hemichannel complexed to OG detergent micelle (nm^3)	Connexin type	Subunit ratio	Theoretical volume of hemichannel (nm^3)	Theoretical volume of hemichannel complexed to OG detergent micelle (nm^3)
Cx26-HA	6:0	335	463	Cx30-HA	6:0	383	511
Cx26-HA/Cx30	5:1	337	465	Cx30-HA/Cx26	5:1	369	497
Cx26-HA/Cx30	4:2	339	467	Cx30-HA/Cx26	4:2	354	482
Cx26-HA/Cx30	3:3	340	468	Cx30-HA/Cx26	3:3	340	468
Cx26-HA/Cx30	2:4	342	470	Cx30-HA/Cx26	2:4	326	454
Cx26-HA/Cx30	1:5	344	472	Cx30-HA/Cx26	1:5	312	440

Table S2. Theoretical molecular volumes, considering all possible stoichiometries, of both heteromeric Cx26-HA/Cx30 and Cx30-HA/Cx26 hemichannels.
